# Willingness to pay for a mosquito bite prevention ‘forest pack’ in Cambodia: results of a discrete choice experiment

**DOI:** 10.1186/s12936-024-05224-2

**Published:** 2024-12-19

**Authors:** Joshua Yukich, Dyna Doum, David J. McIver, Jason H. Richardson, Siv Sovannanoroth, Neil F. Lobo, Allison Tatarsky

**Affiliations:** 1 Tulane University School of Public Health and Tropical Medicine, New Orleans, USA; 2 Health Forefront Organization, Phnom Penh, Cambodia; 3 Malaria Elimination Initiative, Institute for Global Health Sciences, University of California San Francisco, San Francisco, USA; 4 Innovative Vector Control Consortium (IVCC), Liverpool, UK; 5 Cambodia National Centre for Parasitology, Entomology, and Malaria Control, Phnom Penh, Cambodia; 6 Eck Institute for Global Health, University of Notre Dame, South Bend, USA

**Keywords:** Malaria, Greater Mekong Subregion, Spatial repellent, Topical repellent, Willingness to pay, Discrete choice, Vector control, Cambodia

## Abstract

**Background:**

Progress towards malaria elimination in the Greater Mekong Subregion has left much of the residual malaria transmission concentrated among forest-exposed populations for whom traditional domicile focused malaria vector control is unlikely to be effective. New tools to protect these populations from vector biting outdoors are needed.

**Methods:**

Alongside implementation research on the deployment of a “forest pack” consisting of a volatile pyrethroid (transfluthrin)-based spatial repellent (VPSR), a picaridin-based topical repellent and etofenprox treatment of clothing, an assessment was made of participant willingness to pay for the forest packs and variants of the packs using a discrete choice experiment.

**Results:**

Participants showed willingness to pay for forest packs consistent with full-cost recovery for VPSR devices. The inclusion of a full malaria season’s worth of VPSR devices increased the willingness to pay for a forest pack by 15% (*p* = 0.061). At a price of approximately 10 USD, approximately 50% of participants were willing to pay for a forest pack which included a full season’s worth of VPSR.

**Conclusion:**

Forest packs which include VPSR are likely to be acceptable to the target forest-exposed populations, and those which include VPSR products may even have potential for commercial sales or some cost-recovery.

## Background

Between 2019 and 2021, malaria cases declined from over 200,000 cases to less than 70,000 cases across the Greater Mekong Subregion (GMS), including Cambodia [1]. The remaining malaria risk in the region and Cambodia is most concentrated among forest-exposed populations [2–5]. While forest-exposed populations can form a diverse community and reside in different communities within and outside of Cambodia there is a strong association of forest exposure with the logging industry [3].

Malaria control tools that are targeted to indoor feeding and/or resting mosquitoes such as indoor residual spraying and insecticide-treated bed nets may be of limited efficacy in malaria prevention for forest malaria [6, 7]. The expected lack of efficacy of these residence-based interventions is likely due to a combination of human and mosquito behaviours. Firstly, forest-exposed populations are unlikely to reside inside permanent residential structures which can be targeted for indoor residual spraying (IRS), and instead, these groups largely stay in forest camps, sleeping outdoors, in hammocks, or with minimalist, temporary structures for prevention of biting and may move frequently [6]. Additionally, the malaria vector species present in the forested areas of the GMS vary greatly in behaviour and tend to be less endophilic than those in the African settings where most residential vector control tools were developed for use [8].

Malaria vectors in Cambodia have also been documented biting throughout the day [9]. While alternative vector control tools, such as insecticide-treated hammocks, targeted for forest-exposed populations, have been tested and may provide benefits, these tools have not necessarily reached scale in adoption and usage throughout these hard-to-reach groups, especially where hammocks are not already part of the sleeping culture [10–12]. Beyond vector control, other drug-based interventions such as intermittent preventative therapy and targeted drug administration have also been studied and scaled in these populations, but may not be sufficient to eliminate malaria without additional supplemental interventions [13–16].

Using combinations of vector control products targeting outdoor and daytime biting as well as reaching populations and geographic areas not well served by residential interventions may be one way to prevent malaria in places and populations that are incompletely protected by insecticide-treated nets (ITN) or IRS [17–19]. In the Cambodian context, much malaria exposure happens outdoors, especially in the forest and forest fringe [5, 20, 21]. Thus people with exposure to the forest may require targeting for individual mosquito bite prevention strategies in order to reach malaria elimination targets [7, 22–24]. The Cambodian National Malaria Programme initiated the use of “forest packs” in 2018 as part of its intensification plan to accelerate malaria elimination [22]. The national malaria programme sought alternative effective tools for inclusion in the forest pack.

In response, Project BITE (Bite Interruption Toward Elimination) developed and evaluated a new forest pack of bite prevention tools (*BITE pack*), including a volatile pyrethroid spatial repellent (VPSR), topical repellent, and pyrethroid treatment of clothing as one approach to delivering this protection but little is known about personal preferences for the individual components of the pack and willingness to pay (WTP) or accept payment for these products, especially among the populations who potentially stand to benefit most from their use. This manuscript describes the results of a discrete choice experiment (DCE) designed to elicit and quantify preferences and WTP for the forest pack and its component products among a forest-exposed population in Cambodia.

## Methods

Project BITE is a multi-phased research program designed to evaluate the efficacy, acceptability, and feasibility, among other outcomes, of individual and combined bite prevention tools in a forest pack for forest-exposed populations in the GMS. Following a formative assessment and entomological semi-field and field studies, an implementation study was conducted alongside government- and NGO-led distribution of the forest pack in two provinces in Cambodia. As a component of this implementation study, a discrete choice experiment was conducted to estimate willingness to pay for the BITE pack and the desirability of individual components of the BITE pack. While determining the efficacy of the individual components for the BITE pack is outside the scope of this study, other studies within Project BITE have investigated entomological outcomes related to the packs [25].

The BITE pack included a topical repellent (20% picaridin Autan®, SC Johnson), a VPSR (98.68% transfluthrin BiteBarrier™, Pic Corp.), and pyrethroid treatment of clothing (20.3% etofenprox Perimeter Eto Insect Guard™, Pine Belt Processing). The discrete choice experiment included an examination of price levels for the BITE pack and individual components which components are included, and the quantity of each type of product included. The DCE was conducted with a sample of participants who received and had experience with the trialed BITE pack in the larger study. The DCE consisted of choice sets that include the following attributes and levels:

- Price
  - Levels: zero (free), low (20,000 riels), moderate (40,000 riels), high (60,000 riels)

- Topical repellents
  - Levels: not included, 1 month supply for one individual daily use, supply for one individual daily for the duration of the rainy season

- VPSR
  - Levels: not included, 1 month supply for one household, supply for one household for the duration of the rainy season

- Pyrethroid treatment for clothing
  - Levels: not included, treatment for one outfit (shirt and pants/skirt), treatment for all clothing (up to six outfits).

### Study site

The study was conducted with forest-exposed populations in two operational districts (OD): Sen Monorom OD in Mondulkiri province, and Phnom Srouch OD in Kampong Speu province. Both ODs are known *P. falciparum* hotspots and were, therefore, included as part of Project BITE research activities.

### Sample size

A sample size of 100–200 persons was targeted for the DCE survey. Participants were selected from within a larger cross-sectional survey for Project BITE. Given the lack of any prior information on preferences for these products or WTP for them in these settings, a “rule-of-thumb” sample size calculation approach was used [26, 27]. Where minimum sample size can be approximated by the equation $\frac{nta}{c}\ge 500$ where *n* is the number of respondents, *t* is the number of tasks, *a* is the number of alternatives per task, and *c* is the number of levels of the attribute with the largest number of levels. For this study this implied the relation $\frac{n*9*2}{3}\ge 500$ or that the number of respondents needed should be at least 83 persons to ensure the estimation of all first level effects. (Note that price was estimated as a continuous variable and thus is equivalent to having only two levels). Thus, the planned sample size was expected to provide sufficient precision to estimate the demand curves, WTP, and product attribute preferences for the study population.

### Questionnaire design

The script and DCE choice sets were delivered as an independent questionnaire administered to adult participants in the main BITE cross-sectional study during the final data collection round in January and February 2023. The DCE included an introductory script with a practice question/example. This script was followed directly by nine DCE choice sets. The script used to introduce the DCE follows:

### Script

*I am going to ask you a series of questions about the BITE package that you have received from a village health worker or another health worker. These questions are about changes that could be made to the package to understand what might make the package more attractive to you. They also include information about whether or not you would purchase a similar package if it were easily available to you at a shop near your home or your place of work. The questions will present you with two alternative packages (A and B). You will be asked to choose which option you prefer, A or B.*

The questions will follow this form:

*Would you prefer to have package A, which is free, and includes supply of topical mosquito repellent (enough for one person to use daily for the whole rainy season) and pyrethroid treatment of one set of clothing (one shirt and one pair of pants or one skirt), or would you prefer to have package B which will cost 4000 riels and includes a one month supply of a spatial repellent (enough to cover your sleeping area for one month) and pyrethroid treatment of two sets of clothing (two shirts and two pairs of pants or two skirts). The choices are summarized in the table below.*

*Would you prefer option A or option B?*

The questionnaires were implemented in four versions (blocks), one of which was randomly administered to each respondent. Each block contained nine choice sets which were administered to the respondent. The table below shows an example choice set.

An example choice set is shown in Table 1.

**Table 1:** Example choice set

Attribute	A	B
Price	Zero	4000 riels
VPSR	Not included	One month supply
Topical repellent	Full rainy season	Not included
Pyrethroid treatment for clothes	One Set	All clothing

The questionnaire was read aloud to each participant in the Khmer or Bunong language after translation and validation, and participants were shown the table associated with each choice set. Examples of each individual product were presented to participants during the survey for the DCE to ensure that participants were aware of the products they were assessing and to aid in understanding of survey questions.

### Analysis

The socio-demographic features of the participants in the experiment were assessed using general descriptive statistics, and the data from the discrete choice experiment was analyzed using a conditional logit model in which the dependent variable was the choice of whether or not an alternative in a given task was preferred and the predictor variables were the indicator variables for levels of the attributes of the product. The model was estimated conditional on each choice task.

## Results

One-hundred sixty-five people were recruited for the DCE between January 25 and February 8, 2023, in Mondulkiri and Kampong Speu provinces of Cambodia. The sample description is shown in Table 2. All participants in the DCE were responding on behalf of themselves or their minor children and were all involved in activities involving exposure to forested settings, such as working as a forest ranger, farming, or logging. They had all been previously enrolled in the BITE implementation study.

**Table 2:** Sample demographics

Variable	N	Percent
Province	165	
Kampong Speu	50	30%
Mondulkiri	115	70%
Gender	165	
Female	56	34%
Male	109	66%
Age	165	
30 +	114	69%
Under 30	51	31%

The results of the conditional logit model are shown in Table 3. There was no impact of the lowest level of supply of items relative to none for VPSR, topical repellents, and pyrethroid treatment of clothing on the probability of selection of the given forest pack, and as such the low and absent categories were collapsed for the regression. Only a full season’s worth of VPSR significantly improved the probability of choice of an alternative in this setting. Price was associated with an approximate seven percent reduction in the probability of product choice for every 10,000 riels (approximately USD 2.50) increase in price.

**Table 3:** Basic WTP estimates

Term	OR	SE	95% CI Low	95% CI High	p-value
Intercept	1.13	0.09	0.97	1.32	0.120
All clothing treated	1.10	0.09	0.95	1.28	0.217
Full rainy season VPSR	1.16	0.09	0.99	1.35	0.061
Full rainy season topical repellent	1.04	0.08	0.89	1.21	0.616
Price (per 10 k riels	0.93	0.02	0.90	0.96	< 0.001

**Fig. 1:**
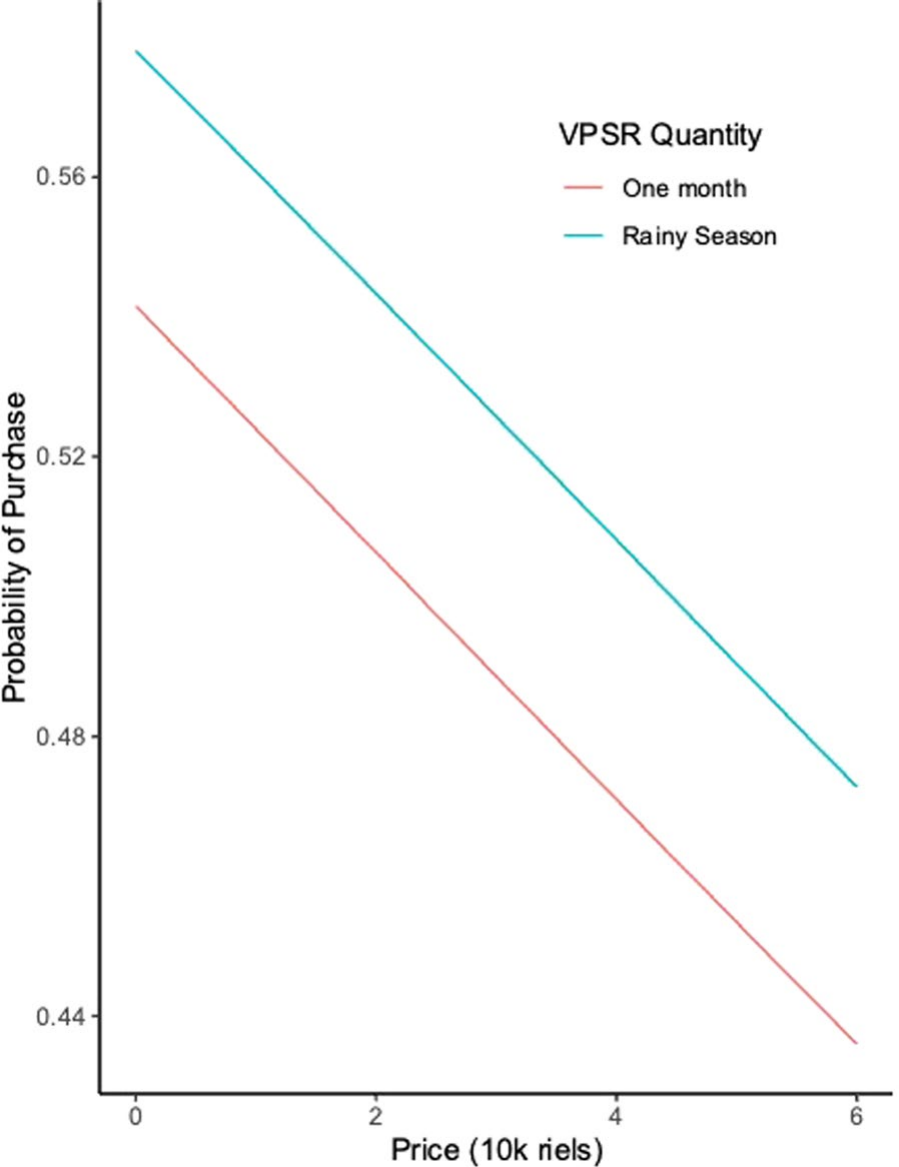
Effect of the price and quantity of Volatile Pyrethroid Spatial Repellent included a forest pack on the probability of purchase of a forest pack

The regression model can be used to project the demand curve for forest packs under various compositions for this population. Figure 1 shows the probability of procurement predicted from the conditional logit model as compared to keeping the purchase price for a product with no or low levels of pyrethroid treatment of clothing or topical repellents at a range of prices included in the study. The probability of procurement is greatly enhanced by including a full season’s worth of VPSR and falls with price.

The WTP regression was also expanded to include individual predictors of WTP, but no available individual factors (age, province of residence, or gender) were significantly associated with willingness to pay for the forest pack or variants (Table 4) in this study. Table 4 shows the results of the WTP conditional logistic regression of individual participants and product characteristics on WTP. Only price had a statistically significant effect at the 5% level. Though inclusion of a larger amount of VPSR product did result in a higher willingness to pay with a *p*-value of approximately 6%.

**Table 4:** Full model WTP estimates

Term	OR	SE	95% CI Low	95% CI High	p-value
Intercept	1.11	0.17	0.80	1.55	0.526
All clothing	1.10	0.08	0.95	1.28	0.217
Full rainy season VPSR	1.16	0.08	0.99	1.35	0.061
Full rainy season topical repellent	1.04	0.08	0.89	1.21	0.617
Price (per 10 k riels)	0.93	0.02	0.90	0.96	< 0.001
Age	1.00	0.00	0.99	1.01	0.955
Male	1.00	0.08	0.86	1.17	0.970
Mondulkiri	1.01	0.08	0.86	1.19	0.909

## Discussion

Forest-exposed populations in Cambodia prefer the VPSR component of forest packs as compared to pyrethroid-treated clothing and topical repellents. The inclusion of the latter two products did not have a statistically meaningful impact on the willingness to pay of participants for the forest packs. A majority of participants reported that they would be willing to purchase a rainy season’s worth of VPSR at a price near 40,000 riel or approximately 10 USD. The demand curves shown in Fig. 1 also indicate that willingness to pay declines meaningfully over the price ranges studied, but that a significant fraction of the population < 30% would not pay for a forest pack even at near-zero prices.

Discrete choice experiments can yield important information about product preferences and utilities. These stated preferences may be useful in guiding product design, marketing, and pricing strategies and could help inform the design of future forest packs to improve uptake and use. The DCE design shows that among this forest-exposed population, preferences for one component of the forest pack (VPSR) dominate all other items except for price in decision.

To our knowledge, this is the first time that a discrete choice method has been used to evaluate preferences for vector control products in Southeast Asia and potentially the first time that this experimental method has been applied to various combinations of vector control tools targeting individual mosquito bite prevention in a near elimination setting.

DCEs such as this one use stated preferences to construct estimates of utility for each component and may differ from the results of revealed preference experiments, such as marketing experiments or auctions when real monetary exchange is involved [28–31]. The results of DCE can be affected by affordability or other practical elements and what is known as hypothetical bias may be introduced when stated preferences are used [32, 33]. Hypothetical bias (the difference between stated preferences and revealed preferences or actual behaviour) is most typically seen as stated preference outcomes indicating a higher willingness to pay than when respondents reveal preferences using their own money. The magnitude and importance of such bias is not consistent across stated and revealed preference studies and as such cannot be known in this case [34].

The sample size for this study was relatively small, and while sufficient to estimate the effects and preferences for the individual products in the forest pack, it is not likely that it was sufficiently powered to detect interaction effects between the various product levels. As such, the marginal utility model used to describe willingness to pay in these data was purely linear and additive and no synergistic or antagonistic effects could be described (i.e. questions such as: did the inclusion of VPSR reduce demand for topical repellents? could not be answered in this context). The high utility of the VPSR as compared to other products in the forest pack may indicate that, at least for this population, the inclusion of VPSR could be a requirement for garnering the use of other vector control products even if these other products demonstrate greater epidemiological effects. Future research should also address individuals’ perceptions of effectiveness and reasons for preferences, especially around VPSR, since users’ perception of risk and the modification of risk using one tool (VPSR) could affect the desirability of or use of other products (e.g. it is possible that users perceive VPSR as effective, and therefore might reduce their use of topical repellents when these tools are available). DCE experiments can be useful for indicating that such phenomena may be present but require additional data collection around preferences to fully explain these kinds of behaviour. While the study population is small, and composed of a distinct group which is not generally representative of the broader Cambodian population, they remain the population which is most at risk for malaria in Cambodia and the main target for ongoing bite prevention efforts targeted at supporting malaria elimination. As such, these results, while not necessarily relevant for broad marketing purposes for forest packs, are likely highly relevant to the places, people and contexts to which this intervention would be targeted.

## Conclusions

VPSRs are desirable components of mosquito bite prevention packs for this forest-exposed population in Cambodia. Most of the target population reports some willingness to pay for these components of the forest pack, at least when enough products are provided to last for an entire rainy season. Price is a strong determinant of stated willingness to pay for individual bite prevention products even when these are desirable products in this population. If forest packs are marketed to forest-exposed populations in Cambodia, including VPSR, keeping prices sufficiently low to ensure affordability for the entire rainy season is likely to be critical in the uptake of such products. The inclusion of VPSR in such a pack is likely to be critical to uptake at any price point.

## Data Availability

All data are available upon request.

## Abbreviations

BITE: Bite interruption toward elimination
DCE: Discrete choice experiment
GMS: Greater Mekong Subregion
IRS: Indoor residual spraying
ITN: Insecticide-treated nets
NGO: Non-governmental organization
OD: Operational district
USD: United States dollar
VPSR: Volatile pyrethroid spatial repellent
WTP: Willingness to pay
